# Fischer’s *Plants in folk beliefs and customs*: a previously unknown contribution to the ethnobotany of the Polish-Lithuanian-Belarusian borderland

**DOI:** 10.1186/s13002-017-0149-8

**Published:** 2017-03-23

**Authors:** Monika Kujawska, Piotr Klepacki, Łukasz Łuczaj

**Affiliations:** 1 Institute of Ethnology and Cultural Anthropology, University of Łódź, Lindleya 3/5, 90-131 Łódź, Poland; 2 Institute of Botany, Jagiellonian University, Kopernika 27, 31-501 Kraków, Poland; 3 Department of Botany, Institute of Biotechnology, University of Rzeszów, Werynia 502, 36-100 Kolbuszowa, Poland

**Keywords:** Historical ethnobotany, Lithuania, Poland, Belarus, Medicinal plants

## Abstract

**Background:**

Historical ethnobotanical studies are useful starting points for further diachronic analysis. The aim of this contribution is to present archival data from the Polish-Lithuanian-Belarusian borderland, which were collected by Adam Fischer, a Polish ethnographer from Lviv, in the 1930s. These data were originally gathered for publication in the first part of the *Lexicon of Slavic beliefs and customs,* dedicated to plant uses in traditional Slavonic culture. It was intended to be a joint international enterprise, but was never actually fulfilled.

**Methods:**

In this article we used information from historical Lithuania (the Great Duchy of Lithuania), nowadays a border region between Poland, Lithuania and Belarus. We applied cultural importance indices such as Use Value, Relative Importance value and Sørensen similarity coefficient, in order to compare our data with a western Ukraine data set from the same research framework.

**Results:**

In total, 153 plant taxa were registered as used in peasant culture in the Polish-Lithuanian-Belarusian borderland in the 1930s. The species which achieved the highest Use Values were: *Calendula officinalis*, *Cyanus segetum*, *Helichrysum arenarium*, *Betula* sp., *Prunella vulgaris*, and *Nuphar lutea* or *Lilium* sp. The most salient use categories were medicinal, followed by food and home garden plants. The overall similarity to plants recorded in western Ukraine within the same project of Fischer’s is quite low (46%), which may be explained by the partly different flora found in the regions, and a cultural discontinuity, revealed by the difference in species with the highest UV. Moreover, the field collaborators were different in the two regions and may have paid attention to different cultural spheres of use.

**Conclusions:**

The presented ethnobotanical data are a valuable contribution to the ethnobotany of Eastern Europe as a whole. In particular, the presented list of plants may be a rich source for future studies on the ethnobotany of the Polish diaspora in Lithuania, and diachronic studies in north-east Poland and Belarus.

## Background

### Ethnobotanical studies concerning Lithuania and Belarus

The archival ethnobotanical data presented in this paper correspond to a historically and culturally complex region. In medieval times it formed part of the Great Duchy of Lithuania. Due to political influences and cultural exchange, the former territory of the Grand Duchy of Lithuania was partly polonized. Between the 16^th^ and 18^th^ centuries Poland and Lithuania formed a kind of commonwealth under the same ruler [1]. Currently the areas from which our information comes belong to the north-eastern outskirts of Poland, Belarus and Lithuania.

In contrast to some other Eastern European countries such as Poland, Hungary or Estonia, there have been very few publications concerning the ethnobotany of Lithuania [2]. This is the case, strangely, both for Lithuanians and for the large Polish population inhabiting the territory of present Lithuania [3]. Some published articles only concern the names of plants and their symbolic meaning in Lithuanian folk culture [4–6]. Contemporary researchers attempt to explain this phenomenon by analysing historical processes, especially the modernization process of the young Lithuanian state from the beginning of the 20^th^ century onwards. In this intellectual context there was little interest in recording ethnobotanical or, especially, ethnomedical knowledge. Instead, ethnologists focused on the poetics of folklore, and within the framework of folk medicine the main objects of interest were verbal formulas, associated with charm healing. Such representation of folk medicine, in conjunction with the ritual use of herbs, was the cause of a stereotypical perception of traditional medicine as an irrational practice [7]. Jan Balys (1909–2011), the prominent Lithuanian ethnologist, deepened the chasm of misunderstanding, claiming that “the largest part of folk medicine is based on similarities (analogies) and has no therapeutic relevance” [8]. Moreover, the territory of the Grand Duchy of Lithuania was for centuries within the radius of activity of Polish culture. However, the 19^th^ and 20^th^ century folklorists concentrated exclusively on Slavic peasants inhabiting the former territory of the Polish-Lithuanian Commonwealth. Even from the Polish language publications in Lithuania, however, we can only list the inventory of medicinal plants sold in the annual Midsummer Herbal Market of Vilnius collected in 1920s by the Polish pharmacist and botanist Jan Muszyński [9]. The author counted more than 100 species, and provided their botanical names, uses and folk names. Some information on plant uses contains vague references to Lithuania in old Polish herbals and economic books [10, 11]. However, the Duchy of Lithuania was huge, hence they could refer to the present Belarus (Polesia), or parts of NE Poland (Podlasie). Rostafinski’s questionnaire of 1883 did not provide any ethnobotanical data either, as the volume of letters from Lithuania is missing from the preserved collection of responses to his questionnaire. Thus the information gathered by Fischer contains the most diverse collection of data, especially on the ethnobotany of the Polish ethnic group in the Vilnius area, the present border region between Belarus and Lithuania.

The ethnobotany of Belarus is much better studied than that of Lithuania, but still insufficiently, taking into account its living tradition of plant use. Kazimierz Moszyński, as far back as 1914, wrote that Polesia (present Belarus) was the most attractive Polish territory (in the sense of the former Kingdom of Poland) from the ethnographic point of view [12]. In his book he included several folk botanical domains, such as food, medicinal and home garden plants. However several other studies are worthy of mention prior to his research, especially from the Grodno region, such as those of the Polish writer Eliza Orzeszkowa [13, 14]. Her lists of plants were based on in-depth fieldworks conducted over several spring-summer seasons in the 1880s. Orzeszkowa’s research was followed in the Grodno area by another female pioneer of ethnobotany, Zośka Wieras. Although born in Ukraine and of Polish-Lithuanian descent (she was also fluent in Russian and Ukrainian), she devoted herself to studying Belarusian folklore. In 1924 she published a Belarusian-Polish-Russian-Latin dictionary [15], which contains the names of a few hundred species of plants, based on her own field data and other sources. Polish ethnographer Michal Fedorowski documented the folk medicine of the 19^th^ century in the former eastern borderlands of the Polish Republic (Belarus today). His *Belarusian folks…*(1897) contains an entire chapter dedicated to plants used in folk therapies [16]. The information was supported by herbal specimens stored in the Warsaw University Botanical Garden [17]. Other minor works concerning the ethnobotany of Belarus were listed in the work by Łuczaj et al. [18]. These authors compared various materials concerning the use of wild foods in Belarus. These were mainly the unpublished questionnaires of Józef Rostafiński from 1883, as well as a handwritten monograph of Michał Fedorowski from the same period [16], as well as contemporary field work data by some of the co-authors of the paper. The contemporary studies dedicated to wild plants used as food, medicine and for animal wellbeing were performed recently in Liubań district, Minsk region by Sǒukand and collegues [19].

### The contribution of Adam Fischer: plants in folk beliefs and customs

Adam Robert Fischer (1889–1943) was a Polish philologist, folklorist and ethnographer. From 1924 onwards he was a professor at the Department of Ethnography at the Jan Kazimierz University in Lwów (now Lviv) [20]. Fischer dedicated most of his life to the development of the Polish Ethnological Society and he also spent 33 years working as the editor-in-chief of *Lud* – the oldest Polish ethnological journal [21]. The legacy of Professor Adam Fischer contains a rich collection of articles, books and unpublished materials, which is now owned by the Polish Ethnological Society. It was transported from Lviv to Wrocław after World War II by the professor’s family [22]. The largest part of Fischer’s ethnobotanical material was the result of his taking part in an international project called the *Lexicon of Slavic beliefs and customs.* The idea for this work arose during the I Congress of Slavic Philologists in Prague in 1929. Its first part was to be focused on plants in folk beliefs and customs. In order to accomplish this task, five editors were appointed from five Slavic countries: Christo Vakarelski from Sofia, Veselin Čajkanović from Belgrade, Karel Chotek from Prague, Adam Fischer from Lwów (now Lviv) and Dmitry Konstantinovich Zelenin from Leningrad (now Saint Petersburg). The mastermind of this enterprise was Edmund Schneeweis from Prague [23]. The *Lexicon* was to be published by the Walter de Gruyter editorial house. The progress of compiling the field work has already been described in our previous contribution [24]. Here we merely repeat the research topics that were investigated during the field campaign. They were published in the form of a questionnaire in 1929 [25] and again in 1930 [26]:

1. Local plant names and possible etymologies
2. Practical application and use of plants in everyday life, such as: food, construction material, cloths, dyeing agents, medicines and poisons
3. Plants with special magical powers, plants in love lore, bestowed with extraordinary virtues enabling the user to ascend into the air or to become invisible
4. Plants with symbolic significance in rituals and ceremonies, such as weddings, funerals and “chodzenie z maikiem” [spring custom of walking around a village with green branches, visiting households and singing songs by peasant youth]
5. Plants as decorative motifs present in houses, on cutlery, clothing, embroidery, cutouts and Easter eggs
6. Toys made from plants, e.g. cockerels, pipes, ropes; caps made of rushes, poppers made of elder, necklaces from rowan, fans, straws etc.
7. Plants in stories and folk songs

In addition to this questionnaire, Fischer enclosed an alphabetical list of 260 plant species according to Polish common names, with Latin names in brackets, which could serve as a prompt for field collaborators. In the archive of the Polish Ethnological Society, stored letters from field collaborators may be found, observing that the list could not have been very productive in the course of collecting materials, as peasants did not know the Polish common names for plants in most cases (Wincenty Bandrowski, Archiwum PTL, sygn. 356). The field information was coupled with voucher specimens, which were identified in the Institute of Botany at Jan Kazimierz University and then returned to field workers. Therefore, no specimens were preserved at the Polish Ethnological Society.

**Fig. 1:**
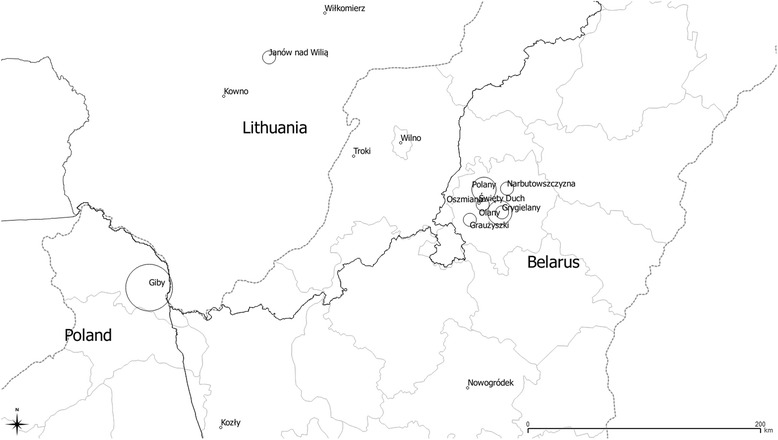
Distribution of the study localities in the 1930s in Polish-Lithuanian-Belarusian borderland. The *dotted line* marks the pre-1939 border of Poland.﻿ *Circles* indicate a size of the dataset from each locality

Fischer was interested in the whole area of pre-World War II Poland, which also includes present western Ukraine and parts of Belarus and Lithuania. He did not manage to publish the results of his research. The aim of this contribution is to describe and analyse a portion of the data set collected for the editing of the *Lexicon*, which corresponds to the historical medieval Lithuania. Nowadays this is NE Poland (Suwałki region), Belarus (Oszmiana area, Polesia region) and Lithuania (Kowno, Troki, etc.) (Fig. 1, Table 1). We also aim to compare this material with the data set from western Ukraine coming from the same period and conducted within the same research framework [24], with regard to the overall composition of species, and medicinal plants in particular.

**Table 1:** Historical and current names of villages and areas studied by Adam Fischer’s field collaborators

Locality	Contemporarty name	Country
Giby	Giby	Poland
Grygielany	Гиpгeляны	Belarus
Janów nad Wilią	Jonava	Lithuania
Kowno (and Kowno area)	Kaunas	Lithuania
Kozły	Кoзлы	Belarus
Narbutowszczyzna	Hapбуты	Belarus
Nowogródek	Haвaгpудaк	Belarus
Graużyszki area	Гpaвжишки	Belarus
Wilno area	Vilnius	Lithuania
Wiłkomierz area	Ukmergė	Lithuania
Olany	Oляны	Belarus
Oszmiana	Aшмяны	Belarus
Polany	Пoляны	Belarus
Święty Duch	Будзёнaўкa	Belarus
Troki	Trakai	Lithuania

## Methods

### Study area

The current borderland between Poland, Belarus and Lithuania used to be part of the Grand Duchy of Lithuania, a European state from the 13^th^ century until 1795, founded by the Lithuanian Baltic tribe. The Grand Duchy, in its expansion, included part of the territories of the present day Belarus, Poland, Ukraine and Russia. The Union of Krewa in 1385 brought two major changes for the Grand Duchy – conversion to Catholicism (it was previously a pagan state) and a dynastic union with the Kingdom of Poland. From 1569 onwards, the union was converted into a Polish-Lithuanian Commonwealth, and Polish became the official language [27]. It was a multi-ethnic, multi-language and multi-religious state. Two macro ethnic groups lived there, the Balts (Lithuanians) and the Slavs (Belarus, Poles, Russians, Ukrainians), as well as smaller groups, such as Jews, Gipsies, Tatars and Karaites [3]. The latter were of Turkic origin, preserving their own language and religion, originating from Judaism [28]. Different religious minorities were encouraged to settle in the Grand Duchy due to the confessional freedom that ruled in the state. Opportunities to practice traditional forms of healing were also probably more favorable in Lithuania in comparison with Western Europe, or Poland, where persecution for witchcraft was widespread [7]. The Russian invasion in 1792 led to the partition of The Duchy between the Russian Empire and Prussia in 1795. After World War I, the Vilnius area and part of Belarus were incorporated into the newly formed Polish Republic as an outcome of the treaty of Riga, local wars and, in case of Vilnius, due to a ruse [1].

### Data collection and botanical identification

Information from the described region was collected during the period between 1929-1935 by several of Adam Fischer’s field collaborators. The field research was conducted in the Polish language among Polish speaking peasants from these territories. We know two field collaborators by name, and the localities in which they conducted their field research (Table 2). However, the number of informants they included in the study remains unknown. Additionally, data came from a manuscript stored in Ossolineum (National Ossoliński Institute) in Wroclaw [29]. These data were mainly from the Janów nad Wilią, Kowno, Troki (nowadays Lithuania).

**Table 2:** Adam Fischer’s field collaborators in the historic Lithuania

Name of field collaborator	Province	County	Locality
Koczorówna Zofia	białostockie	Suwałki	Giby
Koczorówna Zofia	wileńskie	Oszmiana	Narbutowszczyzna
Perls H.	wileńskie	Oszmiana	Grygielany
Perls H.	wileńskie	Oszmiana	Olany
Perls H.	wileńskie	Oszmiana	Polany

**Fig. 2:**
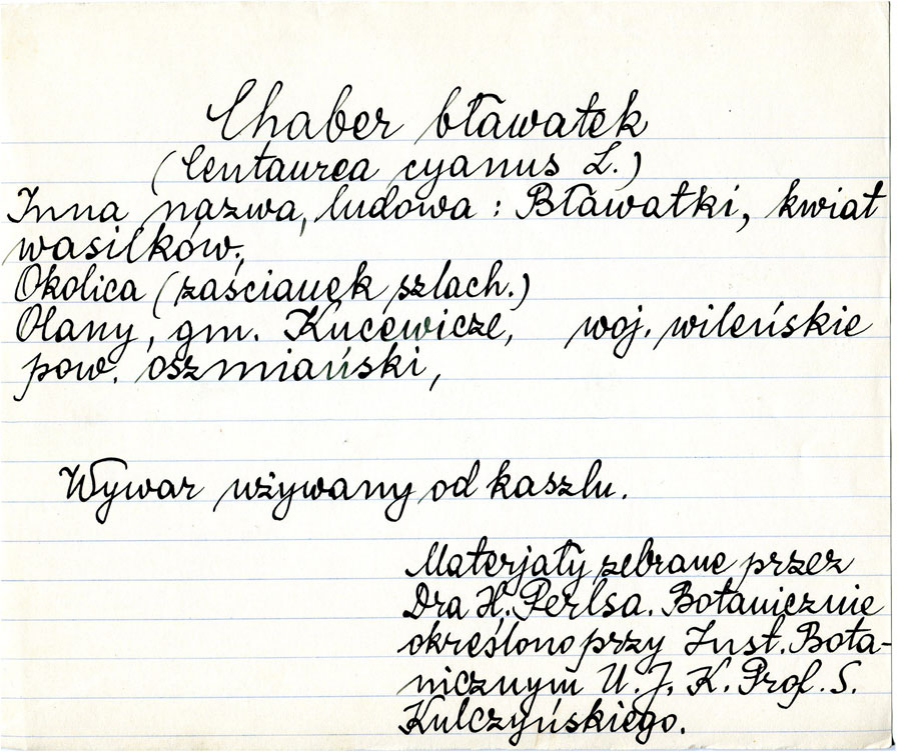
An exemplar filecard with information on plant names, uses and botanical identification from the Vilnius region. [Exact translation: *Chaber bławatek* (Polish vernacular name), followed by Latin name, folk name: *bławatki*, *kwiat wasilików*. Locality: Manor area, Olany, County: Oszmiana, Province: Wilno [Vilnius, now this part is in Belarus]. Use description: decoction used for cough. The information was gathered by H. Perls and botanical material was identified in the Institute of Botany in the University of Jan Kazimierz in Lwów (now Lviv)]

The data set from the Polish-Lithuanian-Belarusian borderland comprises of 283 filecards. Sometimes different cultural uses were lumped together on a single card and sometimes they were split (Fig. 2).

Out of 153 plant taxa whose uses were registered for the study region, nearly all were identified in the Botanical Institute of the Jan Kazimierz University (JKU) in Lwów. However, we do not know if the information recorded in Łaguna’s manuscript underwent botanical identification [29]. All Latin names provided by botanists from the JKU were verified according to the Plant List database [30]. Other Latin names were estimated by the authors, based on common Polish names and local names – as some of the filecards contained both, and some only one of them. The estimated names were then cross-checked with the Polish ethnobotanical and ethnographic literature described in the Background. Cases such as carrot, coriander, caraway and hemp were easy to solve. On several occasions, we were not sure of the correctness of identification, thus we put a question mark (?) next to it. Still, there were some filecards which contained only local names, such as *mieniący kwiat* (*shining flower*), *krowi kwiat* (cow’s flower) and *polska korona* (*Polish crown*), which we could not ascribe to any botanical species – hence these plants and their uses were not included in the analysis.

### Data analysis

A database was used to perform a detailed description of the categories in which plants were used in the Polish-Lithuanian-Belarusian borderland in the 1930s. The material was divided into use categories such as: medicinal, food, household, ritual, veterinary and plants cultivated in home gardens. These were *etic* constructions, which enabled us to apply the Use Value index proposed by Prance et al. [31], and they were modified according to the requirements of archive data. Therefore, in our study each use of a plant taxon was counted as 1.0. Some species had different uses within one category – these were counted as separate uses. Then, for each taxon, we summed up the values corresponding to its uses.

In order to compare the composition of species used in the study region with the data set coming from western Ukraine and collected within the same piece of research, we used the Sørensen similarity coefficient. The formula is as follows: Ss = 2a/(2a + b + c), where a = number of shared species, b = number of exclusive species group 1, c = number of exclusive species group 2. The result is then multiplied by 100, in order to express it as percentage.

The second index of cultural importance we applied was used for medicinal plants only. This was the Relative Importance (RI) value proposed by Bennett and Prance [32], designed to measure medicinal plant versatility. It takes into account two factors: the relative number of body systems (RelBS) treated with a given plant taxon and the relative number of pharmacological properties (RelPH) ascribed to the species. Therefore, this index was appropriate for our set of data, as the number of informants (which is normally required in importance indices) was missing.

## Results and discussion

### General findings

**Fig. 3:**
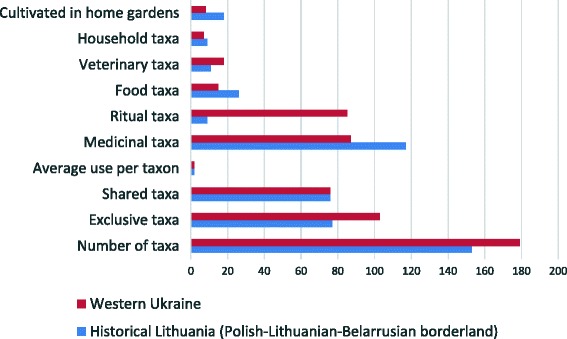
The comparison of taxa and category of uses between Polish-Lithuanian-Belarusian borderland and western Ukraine

In total, 153 plant taxa with 290 uses were still used, or only remembered, in peasant culture in the 1930s in the Polish-Lithuanian-Belarusian borderland (Table 3). The species which achieved the highest Use Value were: *Calendula officinalis* (5), *Cyanus segetum* (5), *Helichrysum arenarium* (5), *Betula* sp. (4), *Prunella vulgaris* (4), and *Nuphar lutea* or *Lilium* sp. (4). Compared to plants used in folk culture in western Ukraine during the same period of time, less species were used (153 and 179, respectively). The most versatile species in the two regions were totally different. According to our previous piece of research, the most versatile plant species in western Ukraine were: *Achillea millefolium* (7), *Allium sativum* (6), *Vinca minor* (6), *Hypericum* sp. (5), *Juniperus communis* (5) [24]. The overall similarity between the two regions is 46%, according to the Sørensen similarity coefficient. This can be explained by the partly different flora found in both regions, and a cultural discontinuity revealed in the difference between species with the highest UV. Moreover, the field collaborators were different in the regions and may have paid attention to different cultural spheres of use. This last observation may explain to some extent the fact that, apart from medicinal plants, which were the most important semantic domains in the two regions, in historical Lithuania a smaller plant richness was registered in domains such as ritual and, veterinary. Especially, the differences in ritual plant uses are striking: 9 species recorded in this study versus 85 spp. in the western Ukraine. In case of food species and plants cultivated in home gardens, more reports were done in the Polish-Lithuanian-Belarusian borderland than in western Ukraine, but the differences are not significant (Fig. 3). However, it should be noted, that the food category is understudied, as some species edible par excellence were recorded only in the medicinal domain: e.g. horseradish (*Armoracia rusticana*) and sorrel (*Rumex* spp.) or carrot reported as medicinal and ritual plant. Therefore, we observe a certain bias towards medicinal plants in the whole set of data from the Fischer’s study, the researchers’ concentration on one domain, which may have influenced the omission of other categories of use, especially those, which were considered quite obvious at that time.

**Table 3:** List of plant species used in folk culture of Polish-Lithuanian-Belarusian borderland

Species	Local name	Category of uses	Use value	Relative importance value
*Achillea millefolium* L.	*krwawnik*	M: bleeding wounds (Giby, Olany, Polany); stomach ache (Olany); V: for cows when they piss with blood (Janów nad Wilią)	3	40
*Achillea ptarmica* L.	*czyszcz*	C: cultivated in home gardens (Giby); R: blessed on Assumption Day (Giby); V: for cows which have just given birth (Giby)	3	-
*Acorus calamus* L.	*koławij*, *francuska trawa*, *tatarak*	F: bread is baked on its leaves (Giby); M: for hair growth (Giby)	2	20
*Aesculus hippocastanum* L.	*kasztan*	M: rheumatism (Olany)	1	20
*Agave* spp. (?)	*oljeander*, *akjaż*	C: cultivated in pots (Giby)	1	-
*Agrostemma githago* L.	*kąkol*	F: stimulant, food seasoning [probably mistaken information] (Giby)	1	-
*Alisma plantago-aquatica* L. (?)	*babka wodna*	M: rabies (Kozły)	1	-
*Allium cepa* L.	*cebula*	M: ulcerous wounds (Giby); women’s genital discharge (Polany)	3	40
*Allium sativum* L.	*czosnek*	M: rheumatism (Giby); intestinal parasites (Polany)	2	40
*Alnus* sp.	*olcha*	H: yellow colour dyeing agent (Giby, Narbutowszczyzna); M: swelling (Giby)	2	20
*Aloe* spp.	*aljas*	M: wounds (Polany)	1	20
*Anchusa officinalis* L.	*miodunka*	M: lung infection (Giby)	1	20
*Angelica* sp.	*dzięgiel*	A: hung in the windows to protect against plague (Troki)	1	-
*Antennaria dioica* (L.) Gaertn.	*no data*	M: folk illness *przełamanie* (Giby); R: blessed on Assumption Day (Giby)	2	20
*Arctium minus* (Hill) Bernh.	*obrymaczki*	M: folk illness fright (Giby); V: swelling in cows (Giby)	2	20
*Arctium tomentosum* Mill.	*rzepnik*	M: folk illness *róża* (Święty Duch)	1	20
*Armoracia rusticana* P. Gaertn., B. Mey. & Schreb.	*chrzan*	M: cough (Narbutowszczyzna); body weakness (Polany)	2	40
*Artemisia abrotanum* L.	*boże drzewko*	C: cultivated in home gardens (Kowno)	1	-
*Artemisia absinthium* L.	*piełunek*, *piołun* (Giby), *pałyn* (Narbutowszczyzna), *piołunek* (Olany)	M: internal pains, general weakness (Giby); stomach ache (Narbutowszczyzna), folk illness *poruszenie* (Olany)	3	60
*Artemisia annua* L. [*A*. *abrotanum* L.]	*boże drzewko*	M: wounds (Polany)	1	20
*Artemisia vulgaris* L.	*bylica*	M: general weakness, folk illness fright (Giby)	2	40
*Astragalus glycyphyllos* L. (?)	*bociany*, *bociani groch*	M: intestinal parasites (Giby)	1	20
*Atriplex* sp.*, Chenopodium* sp.	no data	H: added to fodder improves egg laying (Janów nad Wilią)	1	-
*Avena sativa* L.	*owies*	F: ritual food, porridge during fasting periods (Polany); R: blessed on Assumption Day (Giby)	2	-
*Beta vulgaris* L.	*burok*	F: everyday food, consumed with cabbage and nettles (Narbutowszczyzna)	1	-
*Betula* sp.	*brzoza*	F: bark as famine food added to bread (Narbutowszczyzna); H: leaves as dyeing agent (Narbutowszczyzna); M: kidney infection (Giby); intestinal problems (Olany)	4	40
*Brassica oleracea* L.	*kapusta*	F: everyday food (Narbutowszczyzna); R: blessed on Assumption Day (Giby)	2	-
*Brassica rapa* L.	*rzepa*	F: beverage (Narbutowszczyzna)	1	-
*Briza media* L.	*łezki*, *konopki*	M: kidney infection and urine with blood (Oszmiana)	1	20
*Bryonia alba* L.	*przestęp*	M: wounds, sore throat (Olany)	2	40
*Calendula officinalis* L.	*nagietki* (Olany, Polany, Kowno), *nagietek ogrodowy* (Wiłkomierz area)	C: cultivated in home gardens (Kowno); M: against miscarriage (Olany, Wiłkomierz area); wounds (Wiłkomierz area); V with oatmeal and wheat : for cattle (Olany); for cows in the event of an impact, so as not to calve to early (Polany)	5	40
*Calluna* sp.	*wieroz*	F: seeds as famine food, additive to flour (Narbutowszczyzna)	1	-
*Cannabis sativa* L.	*konopie*	M: ulcerous wounds on head (Giby)	1	20
*Carum carvi* L.	*kmin*	F: fruits as seasoning for bread and cheese (Giby); M: stomach ache, headache (Olany)	3	40
*Centaurium pulchellum* (Sw.) Druce	*centurja*	M: folk illness *poruszenie* (Święty Duch)	1	20
*Centaurium* sp.	*centurja*	M: folk illness *poruszenie* (Polany); liver pain (Grygielany)	2	40
*Chelidonium majus* L.	*złoty groszek*	M: eye infection (Giby)	1	20
*Chenopodium album* L.	*lebioda*	F: famine food as cabbage substitute (Narbutowszczyzna); famine food, eaten boiled with fat (Giby)	1	-
*Chimaphila umbellata* (L.) Nutt.	*stanawnik*	M: folk illness *poruszenie* (Polany)	1	20
*Cichorium intybus* L.	*uraźnik*	M: internal pains, menstrual pains (Giby)	2	40
*Cirsium* sp.*/Carduus* sp.	*oset*	F: everyday food as sorrel substitute, famine food eaten dried (Narbutowszczyzna)	2	-
*Consolida regalis* Gray	*regulka* (Giby), *rahulka* (Święty Duch)	M: colic (Giby, Święty Duch)	1	20
*Convallaria* sp.	*konwalia*	M: internal pains, together with flowers of *Cyanus segetum* (Giby)	1	20
*Convolvulus arvensis* L.	*powojka*	M: general children’s weakness, headache (Giby)	2	40
*Coriandrum sativum* L.	*kolendra*	F: seasoning for meat (Giby)	1	-
*Cyanus segetum* Hill.	*chaber* (Giby, Oszmiana), *wasilki* (Graużyszki, Oszmiana), *bławatki*, *kwiat wasilków* (Olany)	M: eye infection, colic (Giby); ulcerous wounds (Graużyszki); cough (Olany); liquid retention in the organism (Oszmiana)	5	100
*Cynoglossum officinale* L.	*repiej*	M: rheumatism, wounds (Giby)	2	40
*Daucus carota* L.	*marchew*	M: jaundice, drunk together with *Tanacetum vulgare*; R: blessed on Assumption Day (Giby)	2	20
*Dianthus deltoides* L.	*iskraczki*	M: chest pain (Grygielany)	1	20
*Dianthus* sp.	*goździk leśny* (Giby), *goździk* (Kowno)	C: cultivated in home gardens (Kowno); M: intestinal infection (Giby)	2	20
*Elsholtzia ciliata* (Thunb.) Hyl.	*malisa*	M: sore throat (Święty Duch)	1	20
*Epilobium* sp.	*podwiejnik*	M: paralysis, in decoction and smoking; common cold (Oszmiana)	2	40
*Equisetum arvense* L.	*skrzyp*	M: kidney infection, decoction with *Juniperus communis* frutis, *Betula* sp. leaves, and two unidentified plants: *borownik świński*, *koszerka* (Giby)	1	20
*Equisetum pratense* Ehrh.	no data	M: internal illnesses in general (Olany)	1	20
*Euonymus* sp.	*strzmielina*, *ćwiekulec*	H: material for cobbler’s pins (Janów nad Wilią, Wiłkomierz)	1	-
*Ferula* sp.	*smrodzieniec*, *czarcie łajno*	B: lighted reveals sorcerer and expels him (Nowogródek)	1	-
*Fragaria vesca* L.	*poziomka* (Giby), *paziemacznik* (Grygielany), *poziomnik* (Olany)	M: chest pain (Giby, Olany); infertility (Giby); cough (Grygielany)	3	50
*Frangula alnus* Mill.	no data	M: bladder infection (Święty Duch)	1	20
*Geranium palustre* L. (?)	*ślaz*	M: folk illness *poruszenie* (Olany)	1	20
*Glyceria fluitans* (L.) R.Br.	*manna jadalna*	F: seeds as everyday food, type of gruel (Janów nad Wilią)	1	-
*Gymnocarpium dryopteris* (L.) Newman	*urocznik*	M: folk illness fright, evil eye (Olany, Oszmiana, Polany)	2	30
*Helianthemum nummularium* (L.) Mill.	*słonownik*	M: kidney infection (Giby)	1	20
*Helichrysum arenarium* (L.) Moench	*obrymaczki leśne* (Giby), *dramulki* (Grygielany), *miedulki żółte*, *nebot* (Oszmiana)	C: cultivated in pots [may be a mistaken information]; M: lung infection (Giby); abdominal pain (Grygielany); diarrhoea (Oszmiana); R: blessed on Assumption Day (Giby)	5	50
*Hordeum vulgare* L.	*jęczmień*	F: ceremonial food, Christmas eve gruel (Polany)	1	-
*Hypericum perforatum* L.	*świętojańskie ziele*	M: folk illness *poruszenie*, stomach ache (Olany)	2	30
*Inula helenium* L.	*homian* (Janów nad Wilią, Wiłkomierz)	C: cultivated in home gardens (Janów nad Wilią); M: scabies (Wiłkomierz); V: cattle’s broken legs (Janów nad Wilią)	3	20
*Juniperus communis* L.	*jałowiec*	M: respiratory problems, swelling (Polany)	1	40
*Knautia arvensis* (L.) Coult.	*sinowodór*	M: folk illness fright in children (Giby)	1	20
*Lagenaria siceraria* (Molina) Standl. (?)	*tykwa*	C: cultivated in home gardens, H: kept on furniture as adornment (Kowno)	1	-
*Lamium album* L.	*głucha pokrzywa* (Graużyszki), *porzywa biała* (Narbutowszczyzna)	M: heart problems, anaemia (Graużyszki); women’s vaginal discharge (Narbutowszczyzna)	3	50
*Ledum palustre* L.	*rozmaryn leśny*, *bagno*	F: stimulant used as beer adulterant (Wilno area); H: repellent (Wilno area)	2	-
*Leonurus* sp. (?)	*serdecznik biały* (male), *serdecznik czerwony* (female) [there is a belief that two varieties exist : white – male and red – female]	M: internal infections (Giby)	1	20
*Linaria vulgaris* Mill.	*lonnica*	M: smallpox (Graużyszki)	1	20
*Linum usitatissimum* L.	*len*	M: swelling, sore throat (Polany)	2	40
*Lycopodium clavatum* L.	*widlak-dziercza*	M: wounds (Olany)	1	20
*Malus domestica* Borkh.	*jabłoń*	M: heart problems, general weakness (Olany)	2	40
*Malva borealis* Wallman	*ślaz krzaczasty*	M: cough (Giby)	1	20
*Malva* spp.	*ślaz* (Giby, Olany), *różyczka* (Janów nad Wilią)	C: cultivated in home gardens (Janów nad Wilią); M: wounds (Giby); folk illness *poruszenie* (Olany)	3	40
*Matricaria chamomilla* L.	*rumianek*	M: stomach ache (Giby)	1	20
*Melilotus officinalis* (L.) Pall	*borlenin* (?) *żółty* [the local name is difficult to decipher]	M: women’s problems, ulcers, swelling (Giby)	3	60
*Mentha* sp.	*mięta*	C: cultivated in home gardens (Kowno); F: refreshing drink (Olany); M: diarrhoea (Polany)	3	20
*Mentha x piperita* L.	*mięta pieprzowa*	M: bone weakness, vomits (Giby); stomach ache (Olany)	3	50
*Menyanthes trifoliata* L.	*bobek*	M: ague, stomach problems (Olany)	2	40
*Minuartia* sp. (?)	*mokrzyca*	M: swollen limbs (Giby)	1	20
*Nicotiana tabacum* L.	*tytoń*	M: external parasites; decoction harmful to lungs, it can cause death. Men sometimes drink it to avoid being taken to the army (Olany)	2	40
*Nuphar lutea* (L.) Sm.	*łoteć*	M: “Large leaves of this yellow flower put on a wound, as they are cold, they extract fire” (Janów nad Wilią)	1	20
*Nuphar lutea* (L.) Sm. or *Lilium* sp.	*gribilija, żółta lilja*	M: headache, skin infection, folk illness *róża*, women’s vaginal discharge, decoction together with *Lamium album* and *Trifolium repens* (Giby)	4	70
*Origanum vulgare* L.	*lebiodunka*	V: medicine for cattle (Giby)	1	-
*Paeonia* sp.	*piwonja*	C: cultivated in home gardens (Kowno)	1	-
*Papaver somniferum* L.	*mak* (Graużyszki, Polany), *macrek* (Janów nad Wilią)	C: cultivated in home gardens (Janów nad Wilią); F: an ingredient in the Christmas eve dish called *kucja* (Polany); M: toothache (Graużyszki)	3	20
*Papaver* sp.	*maczek*	C: cultivated in home gardens (Kowno)	1	-
*Parnassia cf palustris* L.	*serdecznik żółty*	M: heart problem (Giby, Święty Duch); external ulcers (Oszmiana)	2	40
*Pelargonium grandiflorum* Willd.	*juranim*	M: pneumonia, drunk together with milk and honey (Olany)	1	20
*Persicaria bistorta* (L.) Samp.	*wężownik*	F: famine food, grated leaves are bread additives (Narbutowszczyzna); M: internal pains (Giby)	2	20
*Phlox paniculata* L.	*floks*	C: cultivated in home gardens (Giby)	1	-
*Phragmites australis* (Cav.) Trin. ex Steud.	*trzcina*	A: protects against thunders (Giby); R: blessed on Assumption Day (Giby)	2	-
*Pinus sylvestris* L.	*sosna*	M: tuberculosis (Polany); lung infection (Grygielany, Święty Duch)	2	30
*Pisum sativum* L.	*groch*	H: agrarian knowledge about the best periods to sow the seeds: it says that pea should be sown during the new moon, so it will have a long flowering period, until harvest time. If sown with the north wind, it would be weak, but other people sow it precisely then, because it prevents pea from vermin. (Janów nad Wilią); V: pea, two eggs, a live frog and ink as an excellent remedy for cows which did not moo (Giby)	2	-
*Plantago major* L.	*babka* (Giby), *babka wielka* (Kozły)	M: pains, diarrhoea (Giby); wounds (Kozły)	3	60
*Plantago media* L.	*babka średnia*	M: ague (Kozły); pimples, phlegm and short breathing (Graużyszki)	3	60
*Plantago* sp.	*języczki* (Kozły), *babka* (Polany, Święty Duch)	M: wounds (Kozły, Polany); diarrhoea (Święty Duch)	2	40
*Populus tremula* L.	*osika*	H: house disinfectant (Olany)	1	-
*Potentilla erecta* (L.) Raeusch.	*gałgan*	M: folk illness *poruszenie* (Polany, Święty Duch)	1	20
*Potentilla* sp.	*dzierwanka*, *dzierwianka*	M: lung infection (Olany)	1	20
*Primula veris* L.	*kluczyki*	M: cough (Olany)	1	20
*Prunella vulgaris* L.	*brunelka* (Giby), *dramulki* (Polany), *czemborek* (Graużyszki)	F: refreshing drink, substitute of tobacco (Graużyszki); M: sore throat (Giby, Polany); headache (Giby)	4	40
*Prunus cerasus* L.	*wiśnia*	F: additive to lacto-fermented cucumbers (Polany)	1	-
*Prunus padus* L.	*czeremcha*	M: diarrhoea (Polany)	1	20
*Pteridium aquilinum* (L.) Kuhn (?)	*paprotnik*	F: famine food (Narbutowszczyzna)	1	-
*Pulsatilla* sp.	*sasanka*	M: lung problems (Olany)	1	20
*Quercus* sp.	*dąb*	M: toothache (Polany)	1	20
*Rosa* sp.	*rajska róża*	M: panaceum (Giby)	1	20
*Rubus idaeus* L.	*malina* (Giby), *malinak* (Olany)	M: common cold (Giby); fever (Olany)	2	40
*Rumex* sp.	*koński szczaw*	V: scrofula [horse’s illness] (Janów nad Wilią)	1	-
*Ruta graveolens* L.	*ruta*	C: cultivated in home gardens (Kowno), M: toothache (Giby)	2	20
*Salvia officinalis* L.	*szoławij*	M: sore throat (Giby)	1	20
*Sambucus nigra* L.	*bez czarny*	M: cough (Grygielany, Narbutowszczyzna)	1	20
*Sedum cf acre* L.	*rozchodnik*	M: internal pains (Giby)	1	20
*Sedum maximum* (L.) Suter	*zajęcza kapusta*	M: folk illness *poddźwignięcie*/*ochwat* (Olany)	1	20
*Sempervivum globiferum* L.	*przeskok*	B: used in magic, peasants jump over it (Oszmiana)	1	-
*Sinapis* sp.	*gorczyca*	M: stomach ache, indigestion (Giby, Polany); “one may get retarded out of drinking it” (Giby); V: cows should not eat it, as *gorczyca* may spoil the milk (Giby)	3	40
*Sonchus* sp.	*mleczoj*	M: common cold (Giby)	1	20
*Sorbus aucuparia* L.	*jarzębina*	M: haemorrhoids (Olany), pain in chest (Polany)	2	40
*Succisa pratensis* Moench	*macicznik* (Giby), *naczniczki* (Grygielany), *nizipierśnik* (Olany)	M: uterus problems (Giby); somnifacient for children (Grygielany); ulcers (Olany)	3	60
*Symphyotrichum lanceolatum* (Willd.) G.L.Nesom	no data	C: cultivated in home gardens (Giby)	1	-
*Syringa vulgaris* L.	*bez*	M: cough (Olany), lung infection (Polany)	2	30
*Tagetes* spp. (?)	*aksamitka, maranta*	C: cultivated in home gardens (Kowno)	1	-
*Tanacetum parthenium* (L.) Sch.Bip.	*maruna*	M: indigestion (Giby)	1	20
*Tanacetum vulgare* L.	*wrotycz*	M: jaundice, with carrots [doctrine of signature] (Giby)	1	20
*Taraxacum* sp.	*cykorja polna*	F: everyday food in salads (Lithuania); M: warts (Lithuania)	2	20
*Thymus pulegioides* L.	*macierzanka* (Giby), *czombor* (Olany)	M: panaceum (Giby); lung infection (Olany); R: blessed on Assumption Day (Giby)	3	40
*Tilia cordata* Mill.	*lipa*	M: cough (Olnay); fever (Polany)	2	40
*Tilia* sp.	*lipa*	F: refreshing drink (Polany); M: common cold (Giby); fever (Polany)	3	40
*Trifolium arvense* L.	*koćki*	M: colic (Giby)	1	20
*Trifolium aureum* Pollich	no data	M: jaundice (Polany)	1	20
*Trifolium montanum* L.	*grzmotnik*, *biała koniczyna*, *dzięcielina* (*biała* and *wysoka*)	M: cough (Olany)	1	20
*Trifolium pratense* L.	*koniczyna dzika*	F: refreshing drink (Olany); M: internal problems (Olany)	2	20
*Trifolium repens* L.	*dzięcielinka* (Giby), *koniczyna biała* (Graużyszki)	M: women’s vaginal discharge, used together with *Nuphar lutea* and *Lamium album* (Giby); herpes (Graużyszki)	2	40
*Trigonella foenum-graecum* L.	*kozieradka*	H: to lure wild game (Janów nad Wilią)	1	-
*Tropaeolum majus* L.	*nasturcja*	M: wounds (Polany); infection, together with *Anethum graveolens* and milk (Olany)	2	40
*Tropaeolum* sp.	*naśturcie*	C: cultivated in home gardens (Giby)	1	-
*Tussilago farfara* L.	*podbiał*	M: headache, folk illness *poderwanie* (Giby)	2	40
*Tussilago farfara* L. (?)	*grzybień*	M: cough (Graużyszki)	1	20
*Urtica* spp.	*pokrzywa*	F: everyday food with cabbage and beets (Narbutowszczyzna)	1	-
*Urtica urensi* L.	*rzerzuszka*	M: paralysis, external ulcers, rheumatism (Graużyszki)	3	60
*Vaccinium myrtillus* L.	*jagodnik*	H: black colour dyeing agent (Janów nad Wilią); M: rheumatism (Narbutowszczyzna)	2	20
*Vaccinium vitis-idaea* L.	*brusznicznik*	M: rheumatism (Oszmiana)	1	20
*Valeriana officinalis* L.	*waleriana majowa*	M: neurological problems, anxiety (Oszmiana)	2	20
*Veratrum* sp.	*ciemiężyca*	M: folk illness *poderwanie* (Giby)	1	20
*Verbascum lychnitis* L.	no data	M: stomach ache (Grygielany)	1	20
*Verbascum* sp.	*dziewanna*	M: healing baths (Giby); chest pain (Narbutowszczyzna); V: cattle parasites (Giby)	3	40
*Viola arvensis* Murray	*żółte bratki*, *powojeza*? [a name difficult to decipher]	M: headach, lung infection, healing baths for children (Giby)	3	60
*Viola tricolor* L.	*bratczyki*	M: ulcers (Grygielany)	1	20

Use categories: *A* apotropaic, *B* beliefs, *C* cultivated in home gardens, *F* food, *H* household, *M* medicinal, *R* ritual, *V* – veterinary

The comparison of information collected in Giby village (71 taxa) – nowadays Poland with the rest of the study area (111 taxa) – nowadays Belarus and Lithuania brought quite unexpected results. The overall similarity between the inner two study region (north-east Poland, and nowadays Belarus and Lithuania) is only 35%. Remarkably, all the ritual uses of plants (blessed in church ceremonies) were reported only in Giby. Other culturally important species, with at least three different uses in Giby village, not found in Belarus and Lithuania were *Achillea ptarmica*, *Cichorium intybus*, *Convolvulus arvensis*, *Melilotus officinalis* and *Viola arvensis*. On the other hand, some common and culturally important plants were only reported in Belarus and Lithuania, namely: *Hypericum perforatum*, *Inula helenium*, *Juniperus communis*, *Lamium album*, *Ledum palustre*, *Linum usitatissimum*, *Papaver somniferum*, *Pinus silvestris*, *Sambucus nigra*, *Urtica urens* and *Vaccinium myrtillus*. This analysis leads us to surmise that this data set has its value as a historical material due to the volume of species and the range of an area studied, however the data are too fragmentary to perform any comparison between villages or smaller areas.

The botanical names established by botanists from the Jan Kazimierz University in Lviv are quite reliable. Thirty two taxa were determined to genus level and 121 to species level. However, in the case of nine taxa we put a question mark next to the botanical identification, as we had some doubt about their reliability. These cases are further explained in the Results and Discussion section.

The majority of names are clearly Polish, so we can surmise that the data concerns only Polish-speaking inhabitants. Although Polish names also dominated in the Ukrainian data set, a large number of Ukrainian language names were also recorded [24], in contrast to the present data set which is uniformly Polish.

### Medicinal plants

Plants used in home therapies represent the largest category of uses. We registered 117 taxa and 197 separate uses of plants employed in the treatment of 12 different body systems, of which the most frequently treated were respiratory system disorders (40 plant uses), followed by digestive (33 plant uses) and skin problems (31 uses). A considerable number of plant uses were found in the sub-category of folk illnesses (22 uses). The folk illnesses included evil eye (*uroki*), fright (*przestrach*), *róża* (a skin illness), *poderwanie* (a muscular problem from lifting heavy weights) and *poruszenie* (a digestive problem). They were classified as folk illnesses, as they had no strict equivalent in the biomedical classification of diseases, and normally their treatment also included some magical action apart from using medicinally useful plants. The most frequently treated illnesses in the study region, according to the number of taxa, were: wounds including ulcers and boils (18 different taxa), coughs (10), chest pain (8), stomach ache (8) and headache (6). The species with the highest RI value (the most versatile plants) were: *Cyanus segetum* (100), *Nuphar lutea* (70), *Artemisia absinthium* (60), *Melilotus officinalis* (*idem*), *Plantago major*, *Plantago media*, *Succisa pratensis*, *Urtica urens*, *Viola arvensis*. Moszyński describes in his work *Eastern Polesia* [12], *Nuphar lutea* as a very visible plant in the Polesia landscape and that rhizomes of this species were used in folk treatment, while seeds were eaten by locals.

Medicinal plants were used with greater frequency internally (89 uses) than in the form of external applications (66). We had no data about 42 plant uses, in this respect. The great majority of plants were used solely, and herbal mixtures were reported very rarely. They were only mentioned in the case of remedies against vaginal discharge (*Nuphar lutea*, *Lamium album*, *Trifolium repens*), and jaundice (*Tanacetum vulgare*, *Daucus carota*) – this is also an example of the doctrine of signatures. Infections were treated with *Tropaeolum majus* flowers, seeds of *Anethum graveolens* and milk. Similarly, chest problems were cured with *Pelargonium grandiflorum*, milk and honey. Contemporary studies also confirm that respiratory problems have traditionally been treated with “sweet” remedies [33, 34]. Characteristically, no pharmaceuticals were combined with plant medicines, or at least the field workers did not report this practice.

When we compared medicinal plant species used in this region and western Ukraine (87 taxa) in the 1930s, we found a greater species richness in the Polish-Lithuanian-Belarusian borderland (117 taxa). Only 45 plant species were shared between regions, and the similarity in plant composition reached 44%. The species with the highest RI value were different too, and only *Artemisia absinthium* was shared between the regions. Other most versatile species in western Ukraine were *Achillea millefolium* (100), *Tussilago farfara* (100), *Veratrum album* (87.5), *Allium sativum* (75) and *Viola tricolor* L. (75). Species important in folk medicine in western Ukraine, such as *Matricaria chamomilla,* was recorded with single uses in phytotherapy in historical Lithuania and other important medicinal plants, such as *Arnica montana* were not reported in this study. Many more similarities were found in body systems and illnesses treated with plants, as well as in modes of use and the prevalence of single plant remedies over herbal mixtures [24]. All in all, although peasants in the compared regions used quite different species to treat illnesses, they were used in similar ways, and to treat a similar spectrum of illnesses.

### Other uses of plants

#### Food

This category embraces 26 taxa (29 records). Plants were used in a few subcategories: everyday food (9 records), famine food (7 records), beverages (5 records), spices (3 records), ceremonial food (3 records) and stimulants (2 records). The everyday food subcategory embraces plants used for different purposes. Bread was baked on sweet flag (*Acorus calamus*), and the seeds of another aquatic plant, *Glyceria fluitan,* were collected to make gruel. Cherry leaves were added to pickled cucumbers, and nettle (*Urtica dioica*) was eaten with cabbage (*Brassica oleracea*) and beet (*Beta maritima*). In one of the villages, as a substitute for sorrel (*Rumex* spp.), a wild plant called *oset* (*Cirsium* sp. or *Carduus* sp.) was used. There was one unknown plant called, locally, *legiesie,* which was prepared as lactofermented food. Famine plants are represented by six species. They were used during “war time”, which may be ascribed to World War I or/and the Polish-Soviet war in 1920. These were *oset* (*Carduus* sp. or *Cirsium* sp.), *paprotnik* (*Pteridium aquilinum*?) and bistort (*Persicaria bistorta*). Lamb’s quarters (*Chenopodium album*), common heather (*Calluna vulgaris* – seeds ground and added to flour) and even small pieces of birch wood (*Betula* sp.) were used during food shortages. Five plants were used as herbal teas: mint (*Mentha* sp.), lime flowers (*Tilia* sp.) but also common self-heal (*Prunella vulgaris*), red clover (*Trifolium pretense*) and finally turnip leaves (*Brassica rapa*), which were used in dried form (collected during the spring time) for a drink called *chłodniczek*. In the subcategory of ceremonial food there are only three plants: two used during the Christmas Eve supper: barley (*Hordeum vulgare*, in the form of gruel) and poppy (*Papaver somniferum*, an ingredient for the special Christmas dish *kutia*), and one used during fasting – oats (*Avena sativa*, porridge). Two plants were considered stimulants: common self-heal (*Prunella vulgaris*), whose leaves were smoked, and marsh Labrador tea (*Ledum palustre*), used to adulterate low alcohol beer.

The wild food plant uses in the studied Fischer’s archives contain species already reported from Eastern Europe as wild edible plants used by local population. Some of the rarest uses and those of largest interest is the alimentary use *Pteridium aquilinum* and *Persiacria bistorta*, only documented by Fedorowski [16].

The most intriguing plant in the subcategory of herbal teas is turnip (*Brassica rapa*). We do not know how the tea was used, but the local name of turnip tea *chłodniczek* (means: cooler), suggests its designation. It is the first-noted use of turnip as a beverage plant in this part of Europe [35]. Baking bread on plant leaves was intended to prevent burnt flour from sticking to the fresh bread. Many different species were traditionally used for this purpose – cabbage, oak, maple and sweet flag [36]. This baking additive is still present in the Podlasie region in NE Poland [37]. Famine plants were widely used in Poland, all the listed plants had been noted before [38, 39]. The local name *kąkol* (meaning common corn-cockle) on one of the filecards, with the description “for seasoning food” is probably misleading. *Agrostemma githago,* commonly called *kąkol,* was a well-known toxic plant and a common weed, hence its possible use as a spice is improbable – perhaps a narcotic use should be considered, or maybe some other species was involved.

#### Household

The ‘Household’ category is represented by 9 taxa (15 use reports), and could be distributed into the following subcategories: dyeing (7), animal wellbeing (2), repellents (2), shoemaking (2), hunting (1) and agricultural knowledge (1). Plant-derived dyeing agents were as follows: blueberry (*Vaccinium myrtillus*) for black, with mud also used in the process. Yellow was made by using alder bark or mature “cones” (*Alnus* sp.), as well as birch leaves (*Betula* sp.), with alum as a mordant. The case of a plant called *miczan*, a black dyeing agent, remains unresolved due to the lack of a Latin name in the original data set. Dyeing fabrics with plants has a long history, and it is worthy of note that even during the industrial era, plant dyeing agents were still in use in Poland and adjacent areas [40]. *Błoto* (mud) used in the dyeing process could mean copper sulfate [41]. Another mordant – alum used with birch leaves, was also common [41].

Repellents were prepared out of *Ledum palustre*, which protected cereals in the granary, and aspen bark (*Populus tremula*), mixed with rye flour, was used for house disinfection, removing parasites. *Ledum palustre,* easily available in the region, and used as a repellent, is still in use in NE Poland [37]. Using a mixture made of rye flower and aspen bark against parasites was practically unknown elsewhere, and presumably ineffective [42]. Probably there was some kind of knowledge or superstition about one or both of these plants behind this idea of parasite management, but unfortunately we do not have any data to compare it with.

Interesting information about fenugreek (*Trigonella foenum-graecum*) was found in the data set – it was used by hunters “to lure game animals”. In Polish ethnobotanical materials hunting is not a common category. The usage of fenugreek mentioned on Fischer’s filecards could mean adding spice to attract wild animals by preparing and leaving it in the forest in special food “balls”. People in Augustów Forest (close to Suwałki) still leave food for wild boar in one specific place as a hunting lure, despite the fact that it is illegal nowadays (unpublished materials, P.K.). One piece of information concerns local knowledge about pea growing. It says that pea (*Pisum sativum*) should be sown during the new moon, so it will have a long flowering period, until harvest time. If sown with the north wind, it would be weak, but other people sow it precisely then, because it prevents pea from vermin.

#### Ritual

Fischer’s archives contain very few records concerning ritual plants from the study area: only 9 species of plants are mentioned as traditionally blessed on Assumption Day (August, 15^th^) in Giby near Suwałki, and the painting of Easter eggs with onion is recorded in two localities. Maybe these traditions were too obvious for the informants, who did not pay enough attention to them. On the other hand the tradition of blessing herbs on Assumption, very rich in southern Poland, involved only a few species in NE Poland (compare [36, 37] with [43, 44] for southern Poland). Within the same data set from western Ukraine, 85 different species were used in the rituals by peasants from the region [24]. A similarly characteristic division between NE Poland and the rest of the country was also found on a map prepared by Moszyński and published in 1935, which presented plants stuck into thatched roofs, walls and the like on Midsummer day (23^rd^ of June). NE Poland (today Lithuania) was characterized by the prevalence of use of aspen, nettle and juniper, whose apotropaic powers could be easily explained – their aim was to do harm to witches, who were believed to be particularly active during Midsummer night. In the rest of the country more numerous species were used, with more obscure apotropaic genesis, such as burdock, mugwort, wormwood and lime tree [45, 46].

#### Veterinary

Within this category 11 different species were recorded. *Achillea millefolium*, *Arctium minus*, *Calendula officinalis*, *Inula helenium*, *Origanum vulgare*, and *Sinapis* sp. were the species used especially in the treatment of cows. One recipe from Giby village mentioned pea (*Pisum sativum*), two eggs, a live frog and ink as an excellent remedy for cows which did not moo. Sorrel (Rumex sp.) was used in the treatment of one particular illness which affected horses, namely scrofula. The afore-mentioned plants had a purely curative character and we did not notice anything like the continuity between fodder and veterinary plants used for animal wellbeing, which was observed in the data set from western Ukraine [24].

#### Plants cultivated in home gardens

Home garden plants are represented by 18 species, mainly ornamental: peony (*Peonia* sp.), marigold (*Calendula officinalis*), *Tagetes* spp., mallow (*Malva* sp.), garden nasturtium (*Tropaeolum majus*), perennial phlox (*Phlox paniculata*), panicled aster (*Symphyotrichum lanceolatum*) and sneezewort (*Achillea ptarmica*). Two species were mentioned as potted plants – agave (*Agave* sp.) and, surprisingly – dwarf everlast (*Helichrysum arenarium*). Gourd (*Lagenaria siceraria*) was planted for its fruits, which then were used indoors – mounted on wardrobes and tile stoves for ornamental purposes. This group embraces valuable and useful plants, which could simultaneously be treated as ornamental and medicinal: mint (*Mentha* spp.), rue (*Ruta graveolens*), poppy (*Papaver* sp.), elecampane (*Inula helenium*) and southernwood (*Artemisia abrotanum*).

In the light of the abundance of plant species described from historical Lithuania in the 18^th^ and 19^th^ centuries as common garden plants [11, 47], Fischer’s list is rather short and must have been collected randomly. Mentioning dwarf everlast (*Helichrysum arenarium*) as a potted plant seems to be a mistake. It is a native and common plant in Lithuania and Belarus, requiring well-drained soil with sand and gravel, typical in a postglacial landscape. There is no need to plant it in a pot, as it is easily accessible in the wild or can be cultivated in the garden. The fruits of gourd, Polish *tykwa,* were presumably *Lagenaria siceraria* fruits used for interior decoration. Wyżycki [11] described how to process gourd, to preserve it for long time, which suggests that its use was common long before Fischer’s research.

## Conclusions

The presented ethnobotanical data constitute a valuable contribution to the ethnobotany of Eastern Europe as a whole, however due to their versatility of information they are less likely to lead to a conclusive synthesis. The presented list of plants and the reference list may however be rich sources for future studies of the ethnobotany of the Polish diaspora in Lithuania and diachronic studies in north-east Poland and Belarus.

## Abbreviations

PTL: Polskie Towarzystwo Ludoznawcze (Polish Ethnological Society)

## References

[1] Davies N (1997). Europe: A history.

[2] Petkeviciute Z, Savickiene N, Savickas A, Bernatoniene J, Simaitiene Z, Kalveniene Z, Lazauskas R, Mekas TA (2010). Urban ethnobotany study in Samogitia region, Lithuania. J Med Plant Res.

[3] Engelking A, Golachowska E, Zielińska A (eds.). Tożsamość, Język, Rodzina. Z badań na pograniczu słowiańsko-bałtyckim. Warszawa: Slawistyczny Ośrodek Wydawniczy; 2008.

[4] Šeškauskaitė D, Gliwa B (2002). Rūtà, die Nationalblume der Litauer: Zur Kulturgeschichte der Weinraute (Ruta graveolens L.) und zur Etymologie von litauisch rūtà und deutsch Raute. Anthropos.

[5] Šeškauskaitė D, Gliwa B (2006). Some Lithuanian ethnobotanical taxa: a linguistic view on Thorn Apple and related plants. J Ethnobiol Ethnomed.

[6] Seskauskaitė D, Gliwa B (2010). The Botanical Identity and Cultural Significance of Lithuanian Jovaras: An Ethnobotanical Riddle. Ethnobotany in the new Europe: people, health, and wild plant resources.

[7] Petkevičius R, Typek J, Bilek M (2014). Jan Kazimierz Muszyński (1884- 1957) prekursorem badań etnobotanicznych na Litwie. Etnobiologia Polska.

[8] Balys J. Liaudies magija ir medicina [Folk magic and folk medicine]. Bloomington: Author; 1951.

[9] Muszyński J. Wileńskie zioła ludowe. Wiadomości Farmaceutyczne 1927;21-22:469-476.

[10] Syreński S. Zielnik Herbarzem z ięzyka Łacinskiego zowią To iest Opisanie własne imion, kształtu, przyrodzenia, skutkow, y mocy Zioł wszelakich Drzew Krzewin y korzenia ich, Kwiatu, Owocow, Sokow Miasg, Zywic y korzenia do potraw zaprawowania Takze Trunkow, Syropow, Wodek Lekiwarzow, Konfektow […]. Kraków: W Drukarni Bazylego Skalskiego; 1613.

[11] Wyżycki GJ. Zielnik ekonomiczno-techniczny: czyli opisanie drzew, krzewów i roślin dziko rosnących w kraju, jako też przyswojonych, z pokazaniem użytku ich w ekonomice, rękodziełach, fabrykach i medycynie domowej, z wyszczególnieniem jadowitych i szkodliwych […] ułożony dla gospodarzy i gospodyń. Vol. 2. Wilno: author’s edition; 1845.

[12] Moszyński K (1928). Polesie wschodnie. Materiały etnograficzne z wschodniej części byłego powiatu mozyrskiego oraz powiatu rzeczyckiego.

[13] Orzeszkowa E (1888). Kwiaty i ludzie nad Niemnem. Wisła: miesięcznik geograficzno-etnograficzny.

[14] Orzeszkowa E (1891). Kwiaty i ludzie nad Niemnem. Hommes et plantes des bords du Niemen. Wisła: miesięcznik geograficzno-etnograficzny.

[15] Wieras Z (1924). [Bepac З]. Бeлapуcкa-пoльcкa-paceйcкa-лaцiнcкi бaтaнiчны cлoўнiк.

[16] Fedorowski M (1897). Lud białoruski na Rusi Litewskiej: materyały do etnografii słowiańskiej zgromadzone w latach 1877–1891. T. 1.

[17] Graniszewska M, Leśniewska H, Mankiewicz-Malinowska A, Galera H (2013). *Rośliny użyteczne*… Michała Fedorowskiego – dzieło odnalezione po 130 latach. Etnobiologia Polska.

[18] Łuczaj Ł, Köhler P, Pirożnikow E, Graniszewska M, Pieroni A, Gervasi T (2013). Wild edible plants of Belarus: from Rostafiński’s questionnaire of 1883 to the present. J Ethnobiol Ethnomed.

[19] Sõukand R, Hrynevich Y, Vasilyeva I, Prakofjewa J, Vnukovich Y, Paciupa J, Hlushko A, Knureva Y, Litvinava Y, Vyskvarka S, Silivonchyk H, Paulava A, Kõiva M, Kalle R (2017). Multi-functionality of the few: current and past uses of wild plants for food and healing in Liubań region, Belarus. J Ethnobiol Ethnomed.

[20] Czekanowski J (1946). Półwiecze Towarzystwa Ludoznawczego. Lud.

[21] Jasiewicz Z, Rieszetow AM (2003). Korespondencja między Adamem Fischerem a Dimitrem Konstantynowiczem Zieleninem. Etnografia Polska.

[22] Suchecka P, Jasiewicz Z, Karwicka T (2001). Korespondencja Adama Fischera w Zbiorach Archiwum Naukowego Polskiego Towarzystwa Ludoznawczego. Przeszłość etnologii polskiej w jej teraźniejszości.

[23] Gołąbek J (1929). I Zjazd Filologów Słowiańskich w Pradze. Przegląd Pedagogiczny.

[24] Kujawska M, Łuczaj Ł, Typek J (2015). Fischer’s Lexicon of Slavic beliefs and customs: a previously unknown contribution to the ethnobotany of Ukraine and Poland. J Ethnobiol Ethnomed.

[25] Fischer A (1929). Rośliny w wierzeniach i zwyczajach ludu polskiego. Lud.

[26] Fischer A. Rośliny w wierzeniach i zwyczajach ludu polskiego. Kwestionariusz. Orli Lot. 1930;5-6:85-86.

[27] Łowmiański H (1983). Studia nad dziejami Wielkiego Księstwa Litewskiego.

[28] Dąbrowki G (2013). Prosta etnografia Wileńszczyzny.

[29] Łaguna S. Teki etnograficzne[manuscript nr 4439]. Wrocław: Ossolinum; no date.

[30] The Plant List. 2013. http://www.theplantlist.org/. Accessed 06 Jan 2017.

[31] Prance GT, Balée W, Boom BM, Carneiro RL (1987). Quantitative ethnobotany and the case for conservation in Amazonia. Conserv Biol.

[32] Bennett BC, Prance GT (2000). Introduced plants in the indigenous pharmacopeia of northern South America. Econ Bot.

[33] Paluch A (1984). Świat roślin w tradycyjnych praktykach leczniczych wsi polskiej.

[34] Zamudio F, Kujawska M, Hilgert NI (2010). Honey as Medicinal and Food Resource. Comparison between Polish and Multiethnic Settlements of the Atlantic Forest, Misiones, Argentina. Open Complement Med J Special Issue Med Ethnobiol.

[35] Sõukand R, Quave CL, Pieroni A, Pardo-de-Santayana M, Tardío J, Kalle R, Łuczaj Ł, Svanberg I, Kolosova V, Aceituno-Mata L, Menendez-Baceta G (2013). Plants used for making recreational tea in Europe: a review based on specific research sites. J Ethnobiol Ethnomed.

[36] Gaweł A (2009). Zwyczaje, obrzędy i wierzenia agrarne na Białostocczyźnie od połowy XIX do poczatku XXI wieku.

[37] Klepacki P (2016). Rośliny użytkowe w Puszczy Knyszyńskiej i Beskidzie Niskim. Etnobiologia Polska.

[38] Łuczaj Ł, Szymański W (2007). Wild vascular plants gathered for consumption in the Polish countryside: a review. J Ethnobiol Ethnomed.

[39] Łuczaj Ł (2011). Dziko rosnące rośliny jadalne użytkowane w Polsce od połowy XIX w. do czasów współczesnych. Etnobiologia Polska.

[40] Kowecka E (1963). Farbiarstwo tekstylne na ziemiach polskich (1750-1870). Stud i Mat z Hist Kult Mat.

[41] Mowszowicz J (1985). Przewodnik do oznaczania krajowych roślin zielarskich.

[42] Sõukand R, Kalle R, Svanberg I (2010). Uninvited guests: traditional insect repellents in Estonia used against the clothes moth *Tineola bisselliella*, human flea *Pulex irritans* and bedbug *Cimex lectularis*. Insect Sci.

[43] Łuczaj Ł (2011). Changes in Assumption Day Herbal Bouquets in Poland: a nineteenth century study revisited. Econ Bot.

[44] Łuczaj Ł (2013). Rośliny święcone w bukietach w dniu Matki Boskiej Zielnej w cerkwiach prawosławnych na przedpolu Puszczy Białowieskiej. Plants in bouquets blessed on assumption day in orthodox churches in the vicinity of the Białowieża forest. Etnobiologia Polska.

[45] Moszyński K (1935). Atlas kultury ludowej w Polsce.

[46] Kujawska M. Folk biology of Slavic-speaking peoples. Kazimierz Moszyński and his comparative studies. In: Svanberg I, Łuczaj Ł, editors. Pioneers in European Ethnobiology. Uppsala: Uppsala Studies in Eastern Europe 4; 2014. p. 181–199.

[47] Jundziłł B. Opisanie roślin w Prowincyi Wielkiego Xięstwa Litewskiego naturalnie rosnących według układu Linneusza. Wilno: Drukarnia Pijarów; 1791.

